# Effects of metformin on atrial and ventricular arrhythmias: evidence from cell to patient

**DOI:** 10.1186/s12933-020-01176-4

**Published:** 2020-11-24

**Authors:** Teerapat Nantsupawat, Wanwarang Wongcharoen, Siriporn C. Chattipakorn, Nipon Chattipakorn

**Affiliations:** 1 Division of Cardiology, Department of Internal Medicine, Faculty of Medicine, Chiang Mai University, Chiang Mai, Thailand; 2 Cardiac Electrophysiology Research and Training Center, Faculty of Medicine, Chiang Mai University, Chiang Mai, Chiang Mai, 50200 Thailand; 3 Center of Excellence in Cardiac Electrophysiology Research, Chiang Mai University, Chiang Mai, Thailand; 4 Cardiac Electrophysiology Unit, Department of Physiology, Faculty of Medicine, Chiang Mai University, Chiang Mai, Thailand

**Keywords:** Metformin, Arrhythmias, Atrial fibrillation, Atrial arrhythmias, Ventricular arrhythmias

## Abstract

Metformin has been shown to have various cardiovascular benefits beyond its antihyperglycemic effects, including a reduction in stroke, heart failure, myocardial infarction, cardiovascular death, and all-cause mortality. However, the roles of metformin in cardiac arrhythmias are still unclear. It has been shown that metformin was associated with decreased incidence of atrial fibrillation in diabetic patients with and without myocardial infarction. This could be due to the effects of metformin on preventing the structural and electrical remodeling of left atrium via attenuating intracellular reactive oxygen species, activating 5′ adenosine monophosphate-activated protein kinase, improving calcium homeostasis, attenuating inflammation, increasing connexin-43 gap junction expression, and restoring small conductance calcium-activated potassium channels current. For ventricular arrhythmias, in vivo reports demonstrated that activation of 5′ adenosine monophosphate-activated protein kinase and phosphorylated connexin-43 by metformin played a key role in ischemic ventricular arrhythmias reduction. However, metformin failed to show anti-ventricular arrhythmia benefits in clinical trials. In this review, in vitro and in vivo reports regarding the effects of metformin on both atrial arrhythmias and ventricular arrhythmias are comprehensively summarized and presented. Consistent and controversial findings from clinical trials are also summarized and discussed. Due to limited numbers of reports, further studies are needed to elucidate the mechanisms and effects of metformin on cardiac arrhythmias. Furthermore, randomized controlled trials are needed to clarify effects of metformin on cardiac arrhythmias in human.

## Introduction

Metformin initially received approval from the U.S. Food and Drug Administration for type 2 diabetes in 1995 [1]. Since then, an accumulating body of evidence has shown various benefits of metformin beyond the antihyperglycemic effects [2]. In the case of cardiovascular protection, it has been shown that metformin exerted many benefits including a reduction in blood pressure, left ventricular mass [3], stroke [4], heart failure [5, 6], myocardial infarction (MI), cardiovascular death, and all-cause mortality [7–10]. Several mechanisms behind the cardioprotective effects have been proposed. Metformin is known as 5′ adenosine monophosphate-activated protein kinase (AMPK) activator. Metformin activates AMPK through tyrosine-protein kinase c-Src/phosphatidylinositol-3-kinase (PI3K) pathway activation [11], and/or increased AMP:ATP ratios via inhibition of mitochondrial complex 1 [12]. Once activated, AMPK stimulated endothelial nitric oxide synthase, fatty acid oxidation, glucose transport, glycolysis, cellular calcium handling, ATP-sensitive potassium channels (K_ATP_), autophagy, and inhibited protein synthesis, cell proliferation, endoplasmic reticulum stress, endothelial lipotoxicity, and NF-kB pathway, which helped conserve/generate ATP, prevent necrosis/apoptosis, decrease oxidative stress, decrease inflammation, and prevent atherosclerosis [11, 13].

During ischemic/reperfusion injury, metformin could reduce myocardial infarct size by preserving energy homeostasis via an increase in myocardial adenosine 5′ monophosphate-activated protein kinase (AMPK) activity [14], and stimulating adenosine receptors via increased intracellular formation of adenosine [15]. Once the A1, A2A, A2B, and A3 adenosine receptors in myocardial cells were stimulated, they coupled to G proteins and triggered a range of mostly beneficial responses. These involved activation of protein kinase C, phosphatidylinositol-3-kinase/survival protein kinases (PI3K/Akt), and mitogen-activated protein kinase (MAPK), which ultimately targeted mitochondrial ATP-sensitive potassium (K_ATP_) channels and limited the opening of mitochondrial permeability transition pores (mPTP), leading to protection against necrosis and apoptosis [16–18].

In diabetes-related vasculopathy, metformin was shown to decrease low-density lipoprotein-cholesterol (LDL-C) which may retard the progression of atherosclerosis [19, 20]. However, it has been shown that metformin may not reduce LDL-C, and the anti-atherosclerotic effect of metformin could be independent of lipids-lowering effect [21] and through the improved endothelial function via AMPK [22], downregulation of angiotensin II type 1 receptors, increased antioxidant superoxide dismutase-1 [23], increased cholesterol efflux in macrophages, and decreased plasminogen activator inhibitor type 1 activity, fibrinogen level, C-reactive proteins protein, and NF-kB pathway activation in the vascular wall [24–26]. Heart rate variability, which reflects sympathovagal balance and risk of cardiovascular death in diabetes [27], was also improved following metformin treatment [28].

Despite these cardiovascular benefits of metformin, the roles of metformin on the antiarrhythmic effects are still unclear. In this review, reports regarding the effects and mechanisms of metformin on cardiac arrhythmias are comprehensively summarized and presented. Consistent findings and controversial reports from in vivo and clinical studies are also presented and discussed. This information could provide an important foundation for further work on the benefits of metformin as an antiarrhythmic agent in the future.

## Effects of metformin on atrial arrhythmias: evidence from in vitro and in vivo studies

Atrial fibrillation (AF) is the most common arrhythmia in clinical practice, and has been known for its progressive nature and for heightening the risk of stroke [29]. AF mainly triggered by the pulmonary veins [30] and is perpetuated by multiple wavelets [31, 32] and rotors [33, 34] in the left atrium (LA). Specific stressors, such as heart failure, diabetes, hypertension, obesity, coronary artery disease, aging, or genetic predisposition, have been shown to cause atrial dilatation, interstitial fibrosis, and shortened atrial effective refractory period (AERP) in the LA [29]. An increased atrial pressure in heart failure led to atrial dilatation and fibrosis, which is the structural substrate for AF [35]. It has been demonstrated that insulin resistance and diabetes induced structural, electrical, electro-mechanical, and autonomic remodeling in atria, which subsequently become arrhythmogenic substrates for AF [36]. An increased transforming growth factor-beta (TGF-β), connective tissue growth factor expression, and diastolic dysfunction also led to atrial dilation and fibrosis [36, 37]. An increased L-type calcium current (I_Ca,L_), decreased connexin-43 (Cx43) expression, and reduced sodium current could lead to prolonged action potential duration (APD), increased atrial effective refractory period (AERP) dispersion, and conduction slowing [36, 37]. The combination of conduction delay and atrial fibrosis was shown to lead to excitation-contraction uncoupling [36]. Conversely, AF itself can lead to worsened heart failure due to irregular ventricular filling, loss of atrial contraction, rapid ventricular rates, and tachycardia-induced cardiomyopathy [35]; adverse LA structural remodeling, including myolysis, glycogen deposition, and electrical remodeling, resulting in the promoting of the perpetuation of AF and the setting off of a vicious cycle known as “AF begets AF” phenomenon [38, 39].

AMPK can be activated by metabolic stress and AF, and helps maintain L-type calcium channel current (I_Ca,L_), I_Ca,L_-triggered Ca^2+^ ion transients amplitude, sarcoplasmic reticulum Ca^2+^ content, and cell contractility [40]. Chronicity of AF affects AMPK expression in dogs and humans, with increased AMPK in paroxysmal AF, while paradoxically decreased AMPK in longstanding persistent AF [40–43]. Nonetheless, metformin has been shown to further increase AMPK expression in both situations [41, 42], along with improving insulin resistance, thus it may help prevent atrial arrhythmogenesis.

After rapid atrial pacing in non-diabetic HL-1 atrial cells, metformin was shown to prevent adverse cellular remodeling by attenuating tachy-induced myolysis and reducing intracellular reactive oxygen species (ROS) [44]. In neonatal rat cardiomyocytes, metformin attenuated rapid pacing-induced shortened field potential duration (FPD) by increasing Cx43 gap junction and zonula occluddens-1 (ZO-1) expression via AMPK activation [42]. Not only the direct effects on atrial cells, metformin could improve calcium homeostasis in HL-1 cells by attenuating inflammation of the co-cultured adipocytes via an increased peroxisome proliferator-activated receptor gamma (PPARγ)/adiponectin (APN) and suppressed tumor necrosis factor-alpha (TNFα) [45].

Metformin concentration used in the cell experiments were mostly supra-pharmacological doses. Maximal metformin approved daily dose of 2.5 g results in plasma level of 0.01–0.04 mmol/L [46], while metformin concentration used in the cell experiments ranged from 0.5 to 4 mmol/L [42, 44, 45]. Although low and high metformin concentration can both activate AMPK, the high concentration (> 0.25 mmol/L) also exerted its effects through non-AMPK dependent pathways [46]. Therefore, one should be cognizant when attempting to imply mechanisms and effects of metformin from in vitro reports to clinical studies.

In non-diabetic dogs, rapid atrial pacing (AF model) increased lipid deposition in the left atrial appendages which was associated with AERP shortening and dispersion [41]. These structural and electrical changes are substrates for AF. Administration of metformin for two weeks prior to rapid atrial pacing improved fatty acid β-oxidation via the AMPK/PPAR-α/very long-chain specific acyl-CoA dehydrogenase (VLCAD) signaling pathway, resulting in decreased lipid deposition in the left atrial appendages, and therefore prevented AERP shortening/dispersion. Another study with rapid atrial pacing in dogs showed similar metformin benefits in attenuating shortened AERP, AERP dispersion, and AF reduction via AMPK/Cx43 pathway [42]. Cx43 is the predominant gap junction protein in the heart. AF caused a reduction in atrial Cx43 protein in pigs [47, 48]. Cx43 gene transfer restored atrial Cx43 protein content, improved atrial conduction, and prevented AF [47, 48]. AMPK activation promoted K_ATP_ opening and surface expression, leading to inhibition of gap junction permeability, increase Cx43 expression, and subsequently attenuate atrial arrhythmia [49].

Obesity and diabetes were independently associated with increased risk of new-onset AF. This is partly due to an expansion of epicardial adipose tissue (EAT) under these conditions [50]. EAT is in direct contact with atrial tissues. EAT infiltration and adipokines secreted by EAT could cause atrial inflammation, structural and electrical remodeling, and subsequent AF [51]. Chronic metformin for 6 weeks reduced EAT, inhibited reactive oxygen species (ROS)/NF-kB, decreased pro-inflammatory adipokines (IL-6, TNF-α, and TGF-β1), upregulated adiponectin in LA/EAT, reduced atrial fibrosis, and AF [45].

Small conductance calcium-activated potassium (SK) channels affect cardiac action potential duration during the late-phase repolarization [52]. SK channels are activated by calcium, therefore integrate intracellular calcium changes with membrane potential. SK channels express more in atrial than ventricular myocytes [52]. There are three subtypes, SK1 (K_Ca_2.1), SK2 (K_Ca_2.2), and SK3 (K_Ca_2.3), which are encoded by KCNN1, KCNN2, and KCNN3, respectively [52]. Fu et al. reported an association of SK channels and atrial arrhythmias in diabetic rats [53]. They demonstrated that decreased SK2, increased SK3 expression, distorted current-voltage relationship, and overall SK current reduction in diabetic rats led to prolonged APD and subsequent atrial arrhythmias. Chronic metformin treatment for 3 months reduced atrial arrhythmias by normalizing the APD via an increased SK2, decreased SK3, increased overall SK current, and restored normal current-voltage relationship [53]. Specifically, overexpression of SK3 has been shown to be associated with heart block and atrial arrhythmias [53, 54]. Moreover, the role of SK channels in human AF was reported in genome-wide association analysis, demonstrating an association between single-nucleotide polymorphism in KCNN3 gene with lone AF [52].

**Fig. 1:**
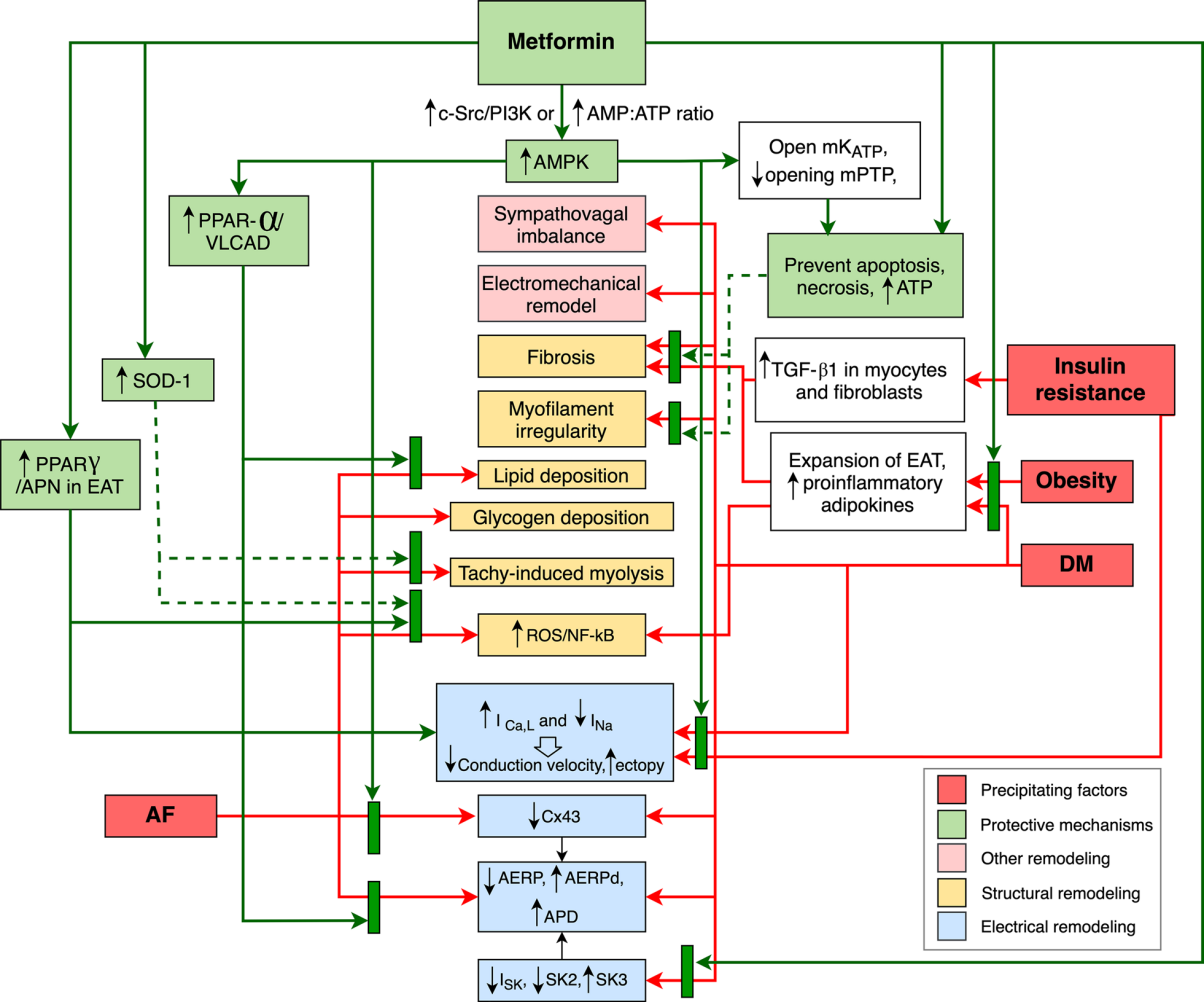
Effects of metformin on atrial arrhythmias. Atrial fibrillation, obesity, insulin resistance, and diabetes mellitus can cause atrial structural, electrical, electromechanical, and autonomic adverse remodeling. These remodelings become arrhythmogenic substrates and set out a vicious cycle known as “AF begets AF”. Metformin exerts protective effects through various mechanisms. Red arrow shows adverse effects from atrial fibrillation, obesity, insulin resistance, and diabetes. Green rectangle shows pathway that metformin blocked. Solid green arrow shows protective mechanisms of metformin directly demonstrated from the studies in Tables 1 and 2. Dotted green arrow indicates protective mechanism of metformin from other studies in the text

In conclusion, rapid atrial pacing induced AF via structural (increased ROS, myolysis, lipid deposition, left atrial fibrosis) and electrical (shortened AERP, increased AERP dispersion) remodeling. Metformin was shown to attenuate this adverse remodeling and break in the “AF begets AF” process. Despite limited in vitro and in vivo reports as summarized in Tables 1 and 2, these in vitro and in vivo studies consistently supported the beneficial effects of metformin on atrial arrhythmias via protection against atrial structural and electrophysiological remodeling in both diabetic and non-diabetic settings. Figure 1 summarized mechanisms behind the protective effects of metformin on atrial arrhythmias.

**Table 1:** Effects of metformin on atrial arrhythmias: reports from in vitro studies

Model	Metformin (dose/ duration)	Key results and major findings							Interpretation	References
		Energy homeostasis	Oxidative stress	Intra-cellular Ca	Inflammation	Cx43	EP changes	Cell structure		
HL-1 atrial cells paced with 4 Hz (240 bpm) for 24 h	1 mmol/L for 2 h	–	↓ROS	–	–	–	–	↑Cytoplasmic myosin heavy chain/nuclear area ratio ↑Troponin I	Metformin provided cardioprotection against AF-related adverse remodeling via attenuating tachy-induced myolysis and oxidative stress of atria cells	[44]
Neonatal rat cardiomyocytes and HL-1 cell with field stimulation at 3 Hz for 12 h	0.5 & 1 mmol/L	↓cAMP→ ↓pSrc→ ↑AMPK→ ↑Cx43	–	–	–	↑	↑FPD	↑ZO-1	Metformin attenuated a shortened FPD possibly by improved gap junction function via AMPK activation, increased ZO-1 and Cx43 expression	[42]
3T3-L1 mature adipocytes with LPS for 24 h then co-cultured with HL-1 atrial cell	4 mmol/L incubated with adipocytes for 12 h	–	–	↓Ca, ↑SERCA2a, ↑pPLN in HL-1 cell	↑PPARγ/ APN, and ↓TNFα in adipocytes	–	–	–	Metformin improved Ca^2+^ homeostasis in HL-1 cell by attenuated the inflammatory interaction between adipocytes and HL-1 cell via an increased PPARγ/APN and suppressed TNFα	[45]

*AMPK* 5' adenosine monophosphate-activated protein kinase, *APN* adiponectin, *Ca* calcium, *cAMP* cyclic adenosine monophosphate, *Cx43* connexin 43, *FPD* field potential duration, *LPS* lipopolysaccharide, *PPARγ* peroxisome proliferator-activated receptor gamma, *pPLN* phosphorylated phospholamban, *p-Src* phopho-Src(Tyr416), *ROS* reactive oxygen species, *SERCA2a* sarcoplasmic reticulum Ca^2+^-ATPase2a, *TNFα* tumor necrosis factor alpha, *ZO-1* Zonula occludens-1

**Table 2:** Effects of metformin on atrial arrhythmias: reports from in vivo studies

Model	Metformin (dose/ duration)	Key results and major findings								Interpretation	References
		Energy homeostasis	ROS	Ion channel	Inflammation	Cx43	EP changes	Structural remodel	AF		
Non-DM dogs with atrial rapid pacing (1200 bpm for 6 h)	100 mg/kg/days for 2 weeks	↑↑AMPK -↑PPARα, PGC-1α, VLCAD, CPT-1	–	–	–	–	↑AERP ↓AERPd	↓FFA/TG /lipid deposition in LAA	–	Metformin improved EP disorders caused by atrial rapid pacing via ↓lipid accumulation and promoted FAO in AF models through AMPK/PPAR-α/VLCAD pathway	[41]
Non-DM dogs with rapid atrial pacing (400 bpm for 6 weeks)	100 mg/kg/days for 1 week prior then 6 weeks	–	↓ in LA/EAT	–	– ↑APN, adipoR1 – ↓ IL-6, NF-kB, TNFα, TGFβ1	–	↑AERP ↓AERPd	↓LA fibrosis and EAT	↓	Metformin reduced AF and atrial fibrosis by inhibited ROS/NF-kB, reduced epicardial fat, pro-inflammatory adipokines, and upregulated adiponectin in LA/EAT	[45]
Non-DM dogs with rapid atrial pacing 400 bpm	100 mg/kg/days for 1 week then pace for 180/ 360 min (acute)	–	–	–	–	↑	↔ AERPd	–	–	Metformin reduced AF by preventing adverse electrical remodeling via increase AMPK and Cx43 expression in chronic AF model. Metformin mildly increased Cx43 in acute pacing and could not attenuate AERPd	[42]
	100 mg/kg/days with pace for 6 weeks (chronic)	↑AMPK ↔ mito-chondrial morphology	–	–	–	↑↑	↑AERP ↓AERPd	↔Irregular myocardial fibers	↓		
GK T2DM rats	300 mg/kg/days for 3 months			- ↔ SK1, ↑SK2,↓SK3 **-**↑SK current **-**↓distorted current-voltage relationship			↓ APD	↓ Atrial myofilament irregularity and fibrosis	↓	Metformin reduced atrial arrhythmia in DM GK rats via decreased atrial remodeling and normalized APD via restoring SK current	[53]

*adipoR1* adiponectin receptor 1, *AERP* atrial effective refractory period, *AERPd* AERP dispersion, *AMPK* 5' adenosine monophosphate-activated protein kinase, *APD* action potential duration, *bpm* beats per minutes, *APN* adiponectin, *Ca* calcium, *CPT-1* Carnitine palmitoyltransferase I, *EAT* epicardial adipose tissue, *EP* electrophysiologic, *FAO* fatty acid oxidation, *FFA* free fatty acid, *GK* Goto-Kakizaki, *LA* left atrium, *LAA* left atrial appendage, *PGC-1α* peroxisome proliferator-activated receptor-gamma coactivator 1α, *PPAR-α* peroxisome proliferator-activated receptor α, *ROS* reactive oxygen species, *SK channels* small conductance calcium-activated potassium channels, *TG* triglyceride, *TGFβ1* transforming growth factor beta1, *TNFα* tumor necrosis factor alpha, *VLCAD* Very long-chain specific acyl-CoA dehydrogenase

## Effects of metformin on atrial arrhythmias: evidence from clinical trials

Observational studies demonstrated that metformin was associated with a reduction in AF incidence, when compared to other anti-diabetic medications, among patients who had diabetes or presented with acute MI [44, 55, 56]. However, the anti-atrial arrhythmias effects seemingly vanished in patients of older age (> 65 years old) or had more advanced diabetes [44, 55, 57]. One study has shown an association between longer DM duration and more advanced atrial remodeling [58]. Older age is known to be associated with more comorbidities (e.g. coronary artery disease, congestive heart failure, hypertension), more fibrous tissue interspersed between myocytes, and electrophysiologic changes of LA [59, 60]. These factors could contribute to the fewer anti-AF effects of metformin seen in these populations. Due to the limitations as an observational cohort, the dose and duration of metformin used in these studies varied and were not reported in detail, thus limiting the analysis of adequacy of dosage and treatment duration.

AF following cardiac surgery occurs not uncommonly with an incidence ranged from 5–64%, and it is associated with prolonged hospital stay, extra cost of care, greater in-hospital mortality, and worse long-term survival [61]. Several mechanisms have been proposed to be accountable for post cardiac surgery AF, including perioperative inflammation, pericarditis, electrical remodeling, autonomic imbalance, atrial incision, perioperative ischemia, and increased oxidative stress [61, 62]. Since metformin has been shown to exert benefits on reducing oxidative stress and inflammation [23, 26, 44, 63–67], it was expected that it might reduce AF in these circumstances. Unfortunately, a randomized controlled trial of 3-day metformin treatment before surgery did not decrease troponin I level or incidence of post cardiac surgery AF in patients without diabetes as compared to placebo [68]. Consistent with this report, metformin was also not associated with decreased post cardiac surgery AF in a retrospective cohort of matched DM patients [69]. Although no in vitro or in vivo studies had directly looked at the performance of metformin on atrial arrhythmias under post cardiac surgery circumstances, these results may imply an ineffectiveness of metformin in preventing AF in post cardiac surgery in the case of both diabetic and non-diabetic patients. All of these reports are summarized in Table 3.

**Table 3:** Effects of metformin on atrial arrhythmias: reports from clinical trials

Model	Type of study/No. of patients/follow-up	Metformin (dose/duration)	AF incidence	Interpretation		References
Taiwanese DM patients treated with metformin alone vs. other meds (mean age 58 years)	Longitudinal cohort/85,198 metformin users and 560,512 non-users/mean follow-up 5.4 years	Various dose and duration of metformin	- ↓ AF incidence in the first 3 years after diagnosis of DM - HR of 0.81, p < 0.0001	Metformin associated with decreased incidence of AF during the first 3 years after the diagnosis of DM		[44]
Taiwanese DM patients w/ or w/o metformin (mean age 69 years)	Nested case control study/2882 AF and 11,528 controls/mean DM duration 3.9 years	- Various dose - At least 6 months of drug used	- ↓ AF incidence - OR 0.81, 95%CI 0.71–0.95	Metformin associated with decreased new onset AF		[55]
Hospitalized DM patients with AMI w/ or w/o metformin (mean age 56)	Retrospective cohort/40 Metformin alone and 705 others/28-day post AMI	Various dose	- Metformin alone: <-> 28-day AF incidence, OR 1.1 (95%CI 0.3–4.0) - Metformin + other anti-DM drugs: ↓28-day AF incidence, OR 0.2 (95% CI 0.1–0.7)	Metformin in combination with other anti-DM drugs associated with decreased AF incidence 28-day post AMI		[56]
≥ 65 years old Taiwanese DM patients w/ or w/o metformin (mean age 73 years)	Nested case-control/1958 cases and 7832 controls/mean DM duration 8 years	- Various dose - At least 12 months of drug used	- <-> AF incidence - OR 1.01 (0.88–1.15)	Metformin was not associated with decreased AF incidence in elderly DM population		[57]
Patients without DM undergoing cardiac surgery randomized to metformin or placebo (mean age 65 years)	Double-blind, randomized controlled trial/57 to metformin, 57 to placebo/24-h post reperfusion	Metformin 500 mg TID for 3 days before surgery	- <-> 24-h post reperfusion Trop I level and arrhythmia	Short-term metformin pre-surgery did not decrease perioperative myocardial injury or AF in non-DM undergoing cardiac surger		[68]
DM pts undergoing cardiac surgery w/ or w/o metformin (mean age 66 years)	Retrospective matched cohort/metformin 635, non-metformin 648/post-op until hospital discharge	Metformin ≥ 500 mg any time prior to surgery	- <-> post-op AF incidence - post-op AF 26.3% in metformin vs. 30.7% in non-metformin(p = 0.46)	Prior use of metformin in DM patients undergoing cardiac surgery was not associated with decreased post-op AF		[69]

*AF* atrial fibrillation, *AMI* acute myocardial infarction, *DM* diabetes mellitus

## Effects of metformin on ventricular arrhythmias: evidence from in vivo studies

Ventricular arrhythmias, which include ventricular tachycardia and ventricular fibrillation (VT/VF), can occur from ischemic and reperfusion (I/R) injury, post-myocardial infarction scar-related reentry, cardiac channelopathy, medication-induced long QT syndrome, or idiopathic [70]. Increased QT interval and QT dispersion reflects prolonged repolarization and inhomogeneity of repolarization, respectively [71]. In diabetes, there are increased corrected QT (QTc) interval and QT dispersion possibly due to alterations in voltage-gated potassium channels [72, 73] and L-type calcium channels [74], and these were associated with a higher risk of sudden cardiac death [75–77]. In animal models, metformin was shown to decrease QT dispersion, and reduce APD and QT interval by inhibiting I_Ca,L _[78, 79]. Post-myocardial infarction ventricular arrhythmias occur from reentry around the scarred and slow-conduction myocardial tissues [70]. Administration of metformin for 2 weeks prior to MI induction in mice could reduce cardiac conduction delay (prolonged PR, QT interval, APD, and conduction velocity), rescue inwardly rectifying potassium channel 2.1 (Kir2.1), and increased Cx43 expression by regulating microRNA-1 overexpression [80]. I/R injury performed in animal studies can largely be divided into 2 models, one with partial occlusion of coronary flow or a non-ST elevation myocardial infarction (NSTEMI) I/R model, and another one with total occlusion of coronary flow or ST elevation myocardial infarction (STEMI) I/R model.

In the STEMI I/R rat model, chronic metformin treatment for 3 weeks has been shown to improve cardiac mitochondrial function, intracellular calcium handling, left ventricular pressure rise (LV dP/dt), and heart rate variability [63]. It also reduced markers for oxidative stress (Malondialdehyde- MDA) and infarct size. However, chronic metformin treatment alone was not able to reduce arrhythmia score or mortality rate [63]. Only when chronic metformin treatment was combined with vildagliptin, could the combination increase phosphorylated Connexin43 (pCX43), and consequently delay time to first VT/VF onset, and reduce arrhythmia score, and mortality rate [63]. In addition, there was no difference of plasma glucose level between the control and the treatment groups, suggesting a direct anti-arrhythmic effect of metformin/vildagliptin beyond an anti-hyperglycemia [63]. These findings are consistent with other studies that showed the importance of the role of pCX43 in the pathogenesis of VT/VF in STEMI models [81–85]. Although studies have been performed in STEMI I/R rodent models which showed the beneficial effects of acute metformin administration (18 h to 2 days prior to ischemia), in reducing infarct size and improving LVEF, arrhythmic outcomes were not measured [14, 86, 87].

In a NSTEMI I/R pig model, acute injection of metformin 3 h prior to ischemia did not provide any benefits regarding AMPK activation, LV dP/dt, electrophysiological changes, and most importantly VT/VF incidence [88]. Unlike acute metformin treatment, chronic metformin treatment for 2–3 weeks in a partial coronary artery occlusion model was shown to reduce VT/VF incidence by preventing monophasic action potential shortening and reducing the dispersion of action potential duration between the ischemic/infarct area and normal myocardium via AMPK activation, leading to preservation of myocardial ATP [88]. The anti-VT/VF effect of metformin was not related to a reduction in blood glucose because it was continuously maintained during the experiments at 4.5 ± 0.5 mmol/L with 10% dextrose solution in both control and treatment groups [88].

**Fig. 2:**
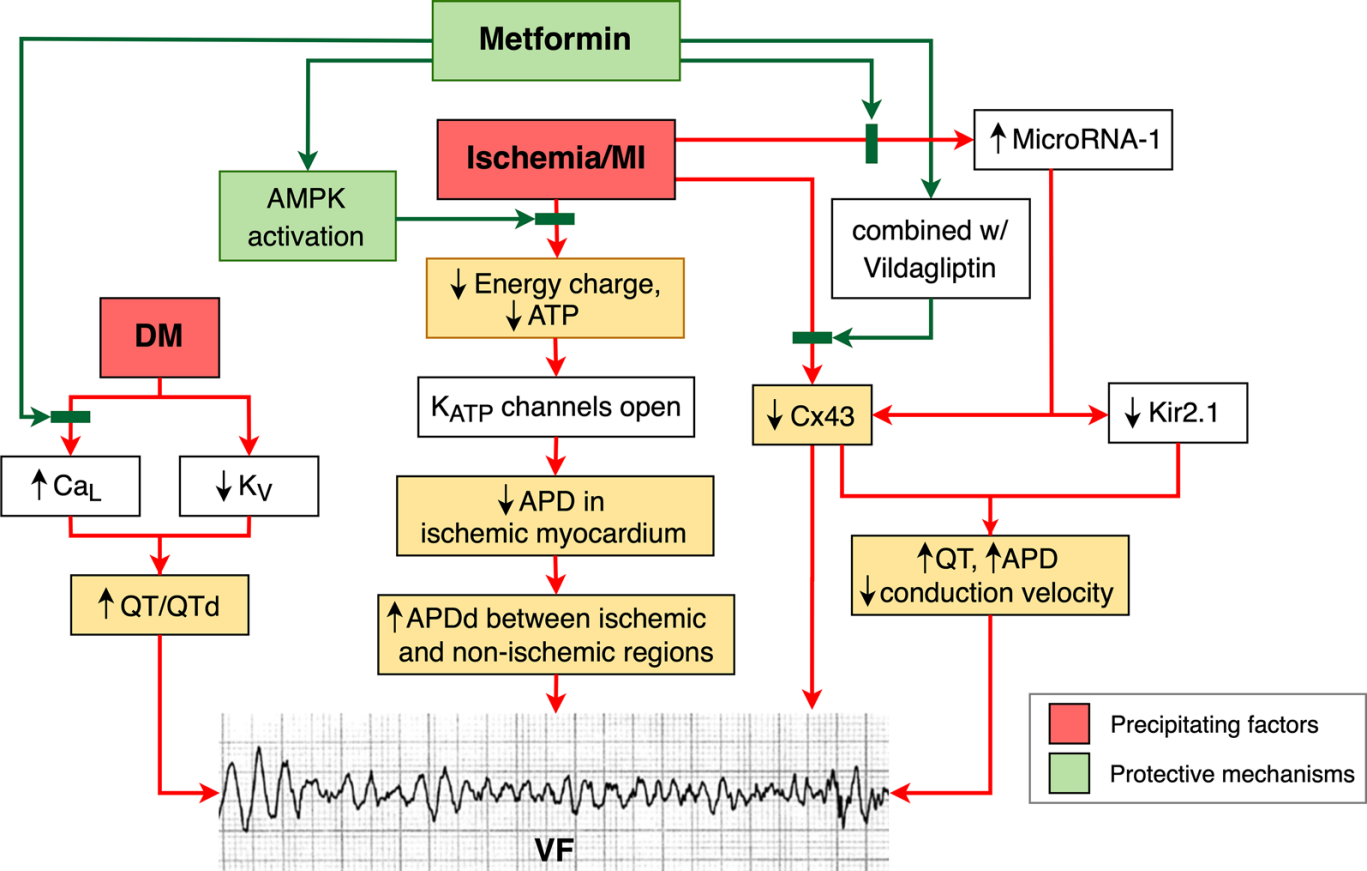
Effects of metformin on ventricular arrhythmias. Ischemia causes reduction in myocardial ATP and finally results in ventricular fibrillation. Chronic metformin use exerts its energy guardian effects mainly via AMPK activation. Additionally, metformin prevents QT interval prolongation, QT dispersion, and conduction velocity delay by regulating microRNA-1 and L-type calcium channels. Only the combination of metformin and vildagliptin could increase p-Cx43 and subsequently reduce ventricular fibrillation. Green rectangle and arrow shows protective mechanisms of metformin

Evidence from these in vivo reports suggested that chronic treatment with metformin alone might reduce VT/VF incidence in the NSTEMI model, whereas the combination of chronic metformin and vildagliptin was required in order to reduce VT/VF in the STEMI model. In contrast, acute metformin treatment did not have any effect on VT/VF events in the NSTEMI model. However, acute metformin treatment was not tested in the STEMI model. All of these reports are summarized in Table 4. Figure 2 summarized mechanisms behind the protective effects of metformin on ventricular arrhythmias.

**Table 4:** Effects of metformin on ventricular arrhythmias: reports from in vivo studies

Model	Metformin (dose/ duration)	Key results and major findings								Interpretation	References
		Energy homeostasis	Oxidative stress	Intra-cellular Ca	LV dP/dt	Infarct/ apoptosis	EP changes	p-Cx 43	VT/ VF		
Domestic farm pigs with cardiac I/R injury -I(50% flow)/R = 90/45 min	-Chronic metformin 30 mg/kg/day per oral for 2–3 weeks)	- ↑↑AMPK - ↑CS - ↑ATP - <-> O_2_, glucose use, lactate	–	–	<->	–	-↓MAP shortening -↓APD dispersion	–	↓	Chronic metformin treatment reduced ischemic VF by preventing MAP shortening and repolarization heterogeneity via AMPK activation, leading to preserved myocardial ATP	[88]
	-Acute Metformin IV 100 mg/kg	<-> AMPK	–	–	<->	–	<->	–	<->		
Male Wistar rats fed with high fat for 12 weeks underwent cardiac I/R injury. (LAD ligation 30/R 120 min)	Metformin 30 mg/kg/day for 3 weeks	↑Mito-chondrial function	↓MDA	-↓Diastolic Ca -↑transient amp/decay	↑	↓Infarct/ ↓Bax, ↑Bcl-2	↑HRV	<->	<->	Metformin alone did not reduce VT/VF incidence. However, combined drugs effectively decreased VT/VF via increased p-Cx43	[63]
	Metformin+ Vildagliptin	↑Mito-chondrial function	↓MDA	-↓Diastolic Ca -↑Transient amp/decay	↑	↓Infarct/ ↓Bax, ↑Bcl-2	↑HRV	↑	↓		

*AMPK* 5' adenosine monophosphate-activated protein kinase, *APD* action potential duration, *ATP* adenosine triphosphate, *Ca* calcium, *CS* citrate synthase, *EP* electrophysiologic, *HRV* heart rate variability, *I/R* ischemic/reperfusion, *LAD* left anterior descending coronary artery, *LV* left ventricular, *MAP* monophasic action potential, *MDA* malondialdehyde, *pAMPK* phosphorylated 5' adenosine monophosphate-activated protein kinase, *p-Cx* phosphorylated Connexin, *VT/VF* ventricular tachycardia/ventricular fibrillation

## Effects of metformin on ventricular arrhythmias: evidence from clinical trials

Patients presented with VT/VF who had diabetes portend worse long-term all-cause mortality at 2 years as compared to patients without diabetes [89]. Hyperglycemia was associated with prolonged QT interval, increased QT dispersion, and higher risk of developing VT in acute MI patients [90, 91]. Whether hyperglycemia is the cause of ventricular arrhythmias, or merely a marker of increased sympathetic activity remains uncertain [92]. Although metformin was associated with a decrease in QTc in diabetic patients [93], there was no available data regarding the relationship between achieving acute hyperglycemic control with metformin/anti-diabetic medications and ventricular arrhythmic outcomes.

There were only two clinical trials that directly studied the effects of metformin on ventricular arrhythmias [56, 94]. In a randomized crossover trial, 19 diabetic patients with coronary artery disease (CAD) were randomized to receive either metformin 500 mg twice daily for 2 weeks or placebo. The primary outcomes were number of premature ventricular contractions/non-sustained VT (PVC/NSVT) beats measured by 24-h Holter monitor [94]. Metformin failed to reduce PVC/NSVT in diabetic CAD patients compared to placebo [94]. However, the results should be interpreted with caution due to the small sample size and a lower-than-average dosage (1000 mg per day) [94] as compared to other studies with cardiovascular benefits (1700–2000 mg per day) [8]. Also, this particular study was not performed under ischemic/reperfusion circumstances, which may explain why metformin did not reveal its anti-ventricular arrhythmia benefits as opposed to the positive findings reported in an animal I/R injury model [88]. Therefore, the PVC/NSVT may be associated with mechanisms other than ischemia, such as automaticity or triggered activity, and might not indicate a poor prognosis.

The second study was a retrospective cohort of hospitalized diabetic patients who presented with acute MI [56]. Unfortunately, metformin was not associated with decreased VT/VF incidence within 28-days post MI [56]. Similar to the first report mentioned above, this report has several limitations, including no data regarding type of MI, unreported metformin dosage and duration, and underutilized beta blocker (23%) and thrombolytic reperfusion therapy (21%). All of these reports are summarized in Table 5.

**Table 5:** Effects of metformin on ventricular arrhythmias: reports from clinical trials

Model	Type of study/No. of patients/FU	Metformin (dose/duration)	Key results and major findings	Interpretation	References
DM patients with CAD monitored via 24-h Holter monitor (mean age 55)	Randomized crossover design/19 patients/2 weeks	Metformin 500 mg BID for 2 weeks	- <-> PVC/NSVT per minute of ischemia	Metformin did not reduce PVC/NSVT in diabetic CAD patients	[94]
Hospitalized DM patients with AMI (mean age 56)	Retrospective cohort/40 Metformin alone and 705 others/28-day post AMI	Various doses	- <-> 28-days VT/VF incidence	Metformin alone or in combination with other anti-DM drugs was not associated with decreased 28-day post AMI VT/VF incidence	[56]

*AMI* acute myocardial infarction, *CAD* coronary artery disease, *DM* diabetes mellitus, *PVC/NSVT* premature ventricular contraction/non-sustained ventricular tachycardia, *VT/VF* ventricular tachycardia/ventricular fibrillation

## Ongoing trials and future research

Two ongoing studies are being carried out regarding the effects of metformin on AF. The first study is a phase 4 randomized open-label study aiming to see whether metformin as compared to placebo could reduce AF burden in patients with paroxysmal or persistent AF who have cardiovascular implantable electronic devices (NCT03603912, TRIM-AF study) [95]. The second study was a phase 2 randomized clinical trial which aimed to see whether metformin could help AF patients stay within a normal sinus rhythm after catheter ablation. This second study had an early termination due to unmet enrollment expectations (NCT02931253) [96]. Unfortunately, there is no ongoing clinical trial of the effects of metformin on ventricular arrhythmias (Table 6).

**Table 6:** Effect of metformin on arrhythmias: ongoing clinical trials

Model	Status	Type of study/No. of patients/FU	Intervention	Primary outcome	References
Patients with paroxysmal or persistent AF with CIED	Recruiting	Phase 4 Randomized clinical trial/270 patients/2 years	- Metformin 750 mg twice daily × 2 years - Lifestyle/risk factor modification	Change in %AF burden at 1 year	[95]
Patients with AF who underwent AF catheter ablation	Terminated	Phase 2 Randomized clinical trial/6 patients/6 months	- Metformin 1000 mg twice daily	Number of patients who maintain sinus rhythm	[96]

*AF* atrial fibrillation, *CIED* cardiovascular implantable electronic device

To progress from the in vivo AF studies, it might be helpful to examine the role of metformin in AF trigger. Since the available reports only assessed AF inducibility and duration after rapid atrial pacing [42, 45], or spontaneous AF detected by surface electrocardiogram [53], this information is not sufficient to determine whether metformin reduced AF by suppressing pulmonary vein triggers or modulating reentry substrate. For in vivo ventricular arrhythmia study, it would be of interest to see whether metformin alone or in combination with a dipeptidyl peptidase-4 inhibitor could reduce ventricular arrhythmias or sudden cardiac death in acute coronary syndrome patients. These hypotheses remain to be elucidated in the future clinical studies.

Although there are some borderline or contradictory results, ample scientific evidence exists to indicate that metformin has potential beneficial effects with regard to atrial and ventricular arrhythmias in human. Adequately-powered randomized controlled trials are needed to clarify the actual effects of metformin both in diabetic and non-diabetic populations. In the case of a study into atrial arrhythmias, use of continuous rhythm monitoring devices, such as an implantable loop recorder, pacemaker, or defibrillator, is strongly encouraged in order to avoid underdetection of AF.

## Conclusions

Basic research has demonstrated the protective effects of metformin on both atrial and ventricular arrhythmias via multiple molecular, cellular, electrophysiological, and structural changes. These findings are mostly translated into anti-atrial arrhythmic benefits seen in clinical trials. However, there are exception in some instances, such as in elderly diabetic or post cardiac surgery patients. Currently, there are very limited clinical reports on the effects of metformin on ventricular arrhythmias and the number of ongoing trials is very small. At this point, proper randomized controlled trials are of the utmost importance in order to clarify the beneficial effects of metformin on cardiac arrhythmias.

## Data Availability

Not applicable.

## Abbreviations

AERP: Atrial effective refractory period
AF: Atrial fibrillation
AMPK: 5′ adenosine monophosphate-activated protein kinase
APD: Action potential duration
APN: Adiponectin
CAD: Coronary artery disease
Cx43: Connexin-43
DM: Diabetes mellitus
EAT: Epicardial adipose tissue
FPD: Field potential duration
I_Ca,L_: L-type calcium current
I/R: Ischemic and reperfusion
K_ATP_ channel: ATP-sensitive potassium channel
LA: Left atrium
LDL-C: Low-density lipoprotein-cholesterol
LV dP/dt: Left ventricular pressure rise
LVEF: Left ventricular ejection fraction
MAPK: Mitogen-activated protein kinase
MDA: Malondialdehyde
MI: Myocardial infarction
mPTP: Mitochondrial permeability transition pores
NSTEMI: Non-ST elevation myocardial infarction
NSVT: Non-sustained ventricular tachycardia
pCX: Phosphorylated connexin
PI3K/Akt: Phosphatidylinositol-3-kinase/survival protein kinases
PPAR: Peroxisome proliferator-activated receptor
PVC: Premature ventricular contraction
QTc: Corrected QT interval
ROS: Reactive oxygen species
SK current: Small conductance calcium-activated potassium current
STEMI: ST elevation myocardial infarction
TGF-β: Transforming growth factor-beta
TNFα: Tumor necrosis factor alpha
VLCAD: Very long-chain specific acyl-CoA dehydrogenase
VT/VF: Ventricular tachycardia and ventricular fibrillation
ZO-1: Zonula occludens-1

## References

[1] Bristol-Myers Squibb Company. Metformin HCL (Glucophage) [package insert]. U.S. Food and Drug Administration website. https://www.accessdata.fda.gov/drugsatfda_docs/nda/pre96/020357Orig1s000rev.pdf. Initial approval 1995.

[2] Rojas LB, Gomes MB (2013). Metformin: an old but still the best treatment for type 2 diabetes. Diabetol Metab Syndr.

[3] Mohan M, Al-Talabany S, McKinnie A, Mordi IR, Singh JSS, Gandy SJ, Baig F, Hussain MS, Bhalraam U, Khan F (2019). A randomized controlled trial of metformin on left ventricular hypertrophy in patients with coronary artery disease without diabetes: the MET-REMODEL trial. Eur Heart J.

[4] Cheng YY, Leu HB, Chen TJ, Chen CL, Kuo CH, Lee SD, Kao CL (2014). Metformin-inclusive therapy reduces the risk of stroke in patients with diabetes: a 4-year follow-up study. J Stroke Cerebrovasc Dis.

[5] Nichols GA, Koro CE, Gullion CM, Ephross SA, Brown JB (2005). The incidence of congestive heart failure associated with antidiabetic therapies. Diabetes Metab Res Rev.

[6] Pantalone KM, Kattan MW, Yu C, Wells BJ, Arrigain S, Jain A, Atreja A, Zimmerman RS (2009). The risk of developing coronary artery disease or congestive heart failure, and overall mortality, in type 2 diabetic patients receiving rosiglitazone, pioglitazone, metformin, or sulfonylureas: a retrospective analysis. Acta Diabetol.

[7] Effect of intensive blood-glucose control with metformin on complications in overweight patients with type 2 diabetes (UKPDS 34). UK Prospective Diabetes Study (UKPDS) Group. *Lancet* 1998, 352(9131):854–865. 9742977

[8] Lamanna C, Monami M, Marchionni N, Mannucci E (2011). Effect of metformin on cardiovascular events and mortality: a meta-analysis of randomized clinical trials. Diabetes Obes Metab.

[9] Han Y, Xie H, Liu Y, Gao P, Yang X, Shen Z (2019). Effect of metformin on all-cause and cardiovascular mortality in patients with coronary artery diseases: a systematic review and an updated meta-analysis. Cardiovasc Diabetol.

[10] Eurich DT, Majumdar SR, McAlister FA, Tsuyuki RT, Johnson JA (2005). Improved clinical outcomes associated with metformin in patients with diabetes and heart failure. Diabetes Care.

[11] Diamanti-Kandarakis E, Christakou CD, Kandaraki E, Economou FN (2010). Metformin: an old medication of new fashion: evolving new molecular mechanisms and clinical implications in polycystic ovary syndrome. Eur J Endocrinol.

[12] Foretz M, Guigas B, Bertrand L, Pollak M, Viollet B (2014). Metformin: from mechanisms of action to therapies. Cell Metab.

[13] Young LH (2008). AMP-activated protein kinase conducts the ischemic stress response orchestra. Circulation.

[14] Solskov L, Lofgren B, Kristiansen SB, Jessen N, Pold R, Nielsen TT, Botker HE, Schmitz O, Lund S (2008). Metformin induces cardioprotection against ischaemia/reperfusion injury in the rat heart 24 hours after administration. Basic Clin Pharmacol Toxicol.

[15] Paiva M, Riksen NP, Davidson SM, Hausenloy DJ, Monteiro P, Goncalves L, Providencia L, Rongen GA, Smits P, Mocanu MM (2009). Metformin prevents myocardial reperfusion injury by activating the adenosine receptor. J Cardiovasc Pharmacol.

[16] Peart JN, Headrick JP (2007). Adenosinergic cardioprotection: multiple receptors, multiple pathways. Pharmacol Ther.

[17] El Messaoudi S, Rongen GA, de Boer RA, Riksen NP (2011). The cardioprotective effects of metformin. Curr Opin Lipidol.

[18] Whittington HJ, Hall AR, McLaughlin CP, Hausenloy DJ, Yellon DM, Mocanu MM (2013). Chronic metformin associated cardioprotection against infarction: not just a glucose lowering phenomenon. Cardiovasc Drugs Ther.

[19] Glueck CJ, Fontaine RN, Wang P, Subbiah MT, Weber K, Illig E, Streicher P, Sieve-Smith L, Tracy TM, Lang JE (2001). Metformin reduces weight, centripetal obesity, insulin, leptin, and low-density lipoprotein cholesterol in nondiabetic, morbidly obese subjects with body mass index greater than 30. Metabolism.

[20] Goldberg R, Temprosa M, Otvos J, Brunzell J, Marcovina S, Mather K, Arakaki R, Watson K, Horton E, Barrett-Connor E (2013). Lifestyle and metformin treatment favorably influence lipoprotein subfraction distribution in the Diabetes Prevention Program. J Clin Endocrinol Metab.

[21] Luo F, Das A, Chen J, Wu P, Li X, Fang Z (2019). Metformin in patients with and without diabetes: a paradigm shift in cardiovascular disease management. Cardiovasc Diabetol.

[22] Mather KJ, Verma S, Anderson TJ (2001). Improved endothelial function with metformin in type 2 diabetes mellitus. J Am Coll Cardiol.

[23] Forouzandeh F, Salazar G, Patrushev N, Xiong S, Hilenski L, Fei B, Alexander RW (2014). Metformin beyond diabetes: pleiotropic benefits of metformin in attenuation of atherosclerosis. J Am Heart Assoc.

[24] Luo F, Guo Y, Ruan GY, Long JK, Zheng XL, Xia Q, Zhao SP, Peng DQ, Fang ZF, Li XP (2017). Combined use of metformin and atorvastatin attenuates atherosclerosis in rabbits fed a high-cholesterol diet. Sci Rep.

[25] Nagi DK, Yudkin JS (1993). Effects of metformin on insulin resistance, risk factors for cardiovascular disease, and plasminogen activator inhibitor in NIDDM subjects. A study of two ethnic groups. Diabetes Care.

[26] Sobel BE, Hardison RM, Genuth S, Brooks MM, McBane RD, Schneider DJ, Pratley RE, Huber K, Wolk R, Krishnaswami A (2011). Profibrinolytic, antithrombotic, and antiinflammatory effects of an insulin-sensitizing strategy in patients in the Bypass Angioplasty Revascularization Investigation 2 Diabetes (BARI 2D) trial. Circulation.

[27] Huikuri HV, Stein PK (2013). Heart rate variability in risk stratification of cardiac patients. Prog Cardiovasc Dis.

[28] Manzella D, Grella R, Esposito K, Giugliano D, Barbagallo M, Paolisso G (2004). Blood pressure and cardiac autonomic nervous system in obese type 2 diabetic patients: effect of metformin administration. Am J Hypertens.

[29] Kirchhof P, Benussi S, Kotecha D, Ahlsson A, Atar D, Casadei B, Castella M, Diener HC, Heidbuchel H, Hendriks J (2016). 2016 ESC Guidelines for the management of atrial fibrillation developed in collaboration with EACTS. Eur Heart J.

[30] Haissaguerre M, Jais P, Shah DC, Takahashi A, Hocini M, Quiniou G, Garrigue S, Le Mouroux A, Le Metayer P, Clementy J (1998). Spontaneous initiation of atrial fibrillation by ectopic beats originating in the pulmonary veins. N Engl J Med.

[31] Cox JL, Canavan TE, Schuessler RB, Cain ME, Lindsay BD, Stone C, Smith PK, Corr PB, Boineau JP (1991). The surgical treatment of atrial fibrillation. II. Intraoperative electrophysiologic mapping and description of the electrophysiologic basis of atrial flutter and atrial fibrillation. J Thorac Cardiovasc Surg.

[32] Moe GK, Abildskov JA (1959). Atrial fibrillation as a self-sustaining arrhythmia independent of focal discharge. Am Heart J.

[33] Haissaguerre M, Hocini M, Denis A, Shah AJ, Komatsu Y, Yamashita S, Daly M, Amraoui S, Zellerhoff S, Picat MQ (2014). Driver domains in persistent atrial fibrillation. Circulation.

[34] Narayan SM, Krummen DE, Shivkumar K, Clopton P, Rappel WJ, Miller JM (2012). Treatment of atrial fibrillation by the ablation of localized sources: CONFIRM (Conventional Ablation for Atrial Fibrillation With or Without Focal Impulse and Rotor Modulation) trial. J Am Coll Cardiol.

[35] Carlisle MA, Fudim M, DeVore AD, Piccini JP (2019). Heart failure and atrial fibrillation, like fire and fury. JACC Heart Fail.

[36] Wang A, Green JB, Halperin JL, Piccini JP (2019). Atrial fibrillation and diabetes mellitus: JACC review topic of the week. J Am Coll Cardiol.

[37] Chan YH, Chang GJ, Lai YJ, Chen WJ, Chang SH, Hung LM, Kuo CT, Yeh YH (2019). Atrial fibrillation and its arrhythmogenesis associated with insulin resistance. Cardiovasc Diabetol.

[38] Casaclang-Verzosa G, Gersh BJ, Tsang TS (2008). Structural and functional remodeling of the left atrium: clinical and therapeutic implications for atrial fibrillation. J Am Coll Cardiol.

[39] Wijffels MC, Kirchhof CJ, Dorland R, Allessie MA (1995). Atrial fibrillation begets atrial fibrillation. A study in awake chronically instrumented goats. Circulation.

[40] Harada M, Tadevosyan A, Qi X, Xiao J, Liu T, Voigt N, Karck M, Kamler M, Kodama I, Murohara T (2015). Atrial fibrillation activates AMP-dependent protein kinase and its regulation of cellular calcium handling: potential role in metabolic adaptation and prevention of progression. J Am Coll Cardiol.

[41] Bai F, Liu Y, Tu T, Li B, Xiao Y, Ma Y, Qin F, Xie J, Zhou S, Liu Q (2019). Metformin regulates lipid metabolism in a canine model of atrial fibrillation through AMPK/PPAR-alpha/VLCAD pathway. Lipids Health Dis.

[42] Li J, Li B, Bai F, Ma Y, Liu N, Liu Y, Wang Y, Liu Q (2020). Metformin therapy confers cardioprotection against the remodeling of gap junction in tachycardia-induced atrial fibrillation dog model. Life Sci.

[43] Qiu J, Zhou S, Liu Q (2016). Energy metabolic alterations in the progression of atrial fibrillation: Potential role of AMP-activated protein kinase as a critical regulator. Int J Cardiol.

[44] Chang SH, Wu LS, Chiou MJ, Liu JR, Yu KH, Kuo CF, Wen MS, Chen WJ, Yeh YH, See LC (2014). Association of metformin with lower atrial fibrillation risk among patients with type 2 diabetes mellitus: a population-based dynamic cohort and in vitro studies. Cardiovasc Diabetol.

[45] Li B, Po SS, Zhang B, Bai F, Li J, Qin F, Liu N, Sun C, Xiao Y, Tu T (2020). Metformin regulates adiponectin signalling in epicardial adipose tissue and reduces atrial fibrillation vulnerability. J Cell Mol Med.

[46] He L, Wondisford FE (2015). Metformin action: concentrations matter. Cell Metab.

[47] Bikou O, Thomas D, Trappe K, Lugenbiel P, Kelemen K, Koch M, Soucek R, Voss F, Becker R, Katus HA (2011). Connexin 43 gene therapy prevents persistent atrial fibrillation in a porcine model. Cardiovasc Res.

[48] Igarashi T, Finet JE, Takeuchi A, Fujino Y, Strom M, Greener ID, Rosenbaum DS, Donahue JK (2012). Connexin gene transfer preserves conduction velocity and prevents atrial fibrillation. Circulation.

[49] Qiu J, Zhou S, Liu Q (2016). Phosphorylated AMP-activated protein kinase slows down the atrial fibrillation progression by activating Connexin43. Int J Cardiol.

[50] Kim YG, Han KD, Choi JI, Boo KY, Kim DY, Oh SK, Lee KN, Shim J, Kim JS, Kim YH (2019). The impact of body weight and diabetes on new-onset atrial fibrillation: a nationwide population based study. Cardiovasc Diabetol.

[51] Packer M (2019). Disease-treatment interactions in the management of patients with obesity and diabetes who have atrial fibrillation: the potential mediating influence of epicardial adipose tissue. Cardiovasc Diabetol.

[52] Zhang XD, Lieu DK, Chiamvimonvat N (2015). Small-conductance Ca2+ -activated K + channels and cardiac arrhythmias. Heart Rhythm.

[53] Fu X, Pan Y, Cao Q, Li B, Wang S, Du H, Duan N, Li X (2018). Metformin restores electrophysiology of small conductance calcium-activated potassium channels in the atrium of GK diabetic rats. BMC Cardiovasc Disord.

[54] Zhang XD, Timofeyev V, Li N, Myers RE, Zhang DM, Singapuri A, Lau VC, Bond CT, Adelman J, Lieu DK (2014). Critical roles of a small conductance Ca(2)(+)-activated K(+) channel (SK3) in the repolarization process of atrial myocytes. Cardiovasc Res.

[55] Liou YS, Yang FY, Chen HY, Jong GP (2018). Antihyperglycemic drugs use and new-onset atrial fibrillation: a population-based nested case control study. PLoS One.

[56] Davis TM, Parsons RW, Broadhurst RJ, Hobbs MS, Jamrozik K (1998). Arrhythmias and mortality after myocardial infarction in diabetic patients. Relationship to diabetes treatment. Diabetes Care.

[57] Chen HY, Yang FY, Jong GP, Liou YS (2017). Antihyperglycemic drugs use and new-onset atrial fibrillation in elderly patients. Eur J Clin Invest.

[58] Dublin S, Glazer NL, Smith NL, Psaty BM, Lumley T, Wiggins KL, Page RL, Heckbert SR (2010). Diabetes mellitus, glycemic control, and risk of atrial fibrillation. J Gen Intern Med.

[59] Anyukhovsky EP, Sosunov EA, Chandra P, Rosen TS, Boyden PA, Danilo P, Rosen MR (2005). Age-associated changes in electrophysiologic remodeling: a potential contributor to initiation of atrial fibrillation. Cardiovasc Res.

[60] Anyukhovsky EP, Sosunov EA, Plotnikov A, Gainullin RZ, Jhang JS, Marboe CC, Rosen MR (2002). Cellular electrophysiologic properties of old canine atria provide a substrate for arrhythmogenesis. Cardiovasc Res.

[61] Mostafa A, El-Haddad MA, Shenoy M, Tuliani T (2012). Atrial fibrillation post cardiac bypass surgery. Avicenna J Med.

[62] Oktay V, Baydar O, Sinan UY, Kocas C, Abaci O, Yildiz A, Yigit Z, Yildiz CE, Hatemi A, Cetin G (2014). The effect of oxidative stress related with ischemia-reperfusion damage on the pathogenesis of atrial fibrillation developing after coronary artery bypass graft surgery. Turk Kardiyol Dern Ars.

[63] Apaijai N, Chinda K, Palee S, Chattipakorn S, Chattipakorn N (2014). Combined vildagliptin and metformin exert better cardioprotection than monotherapy against ischemia-reperfusion injury in obese-insulin resistant rats. PLoS One.

[64] Chakraborty A, Chowdhury S, Bhattacharyya M (2011). Effect of metformin on oxidative stress, nitrosative stress and inflammatory biomarkers in type 2 diabetes patients. Diabetes Res Clin Pract.

[65] Diaz-Morales N, Rovira-Llopis S, Banuls C, Lopez-Domenech S, Escribano-Lopez I, Veses S, Jover A, Rocha M, Hernandez-Mijares A, Victor VM (2017). Does metformin protect diabetic patients from oxidative stress and leukocyte-endothelium interactions?. Antioxid Redox Signal.

[66] Esteghamati A, Eskandari D, Mirmiranpour H, Noshad S, Mousavizadeh M, Hedayati M, Nakhjavani M (2013). Effects of metformin on markers of oxidative stress and antioxidant reserve in patients with newly diagnosed type 2 diabetes: a randomized clinical trial. Clin Nutr.

[67] Malinska H, Oliyarnyk O, Skop V, Silhavy J, Landa V, Zidek V, Mlejnek P, Simakova M, Strnad H, Kazdova L (2016). Effects of Metformin on Tissue Oxidative and Dicarbonyl Stress in Transgenic Spontaneously Hypertensive Rats Expressing Human C-Reactive Protein. PLoS One.

[68] El Messaoudi S, Nederlof R, Zuurbier CJ, van Swieten HA, Pickkers P, Noyez L, Dieker HJ, Coenen MJ, Donders AR, Vos A (2015). Effect of metformin pretreatment on myocardial injury during coronary artery bypass surgery in patients without diabetes (MetCAB): a double-blind, randomised controlled trial. Lancet Diabetes Endocrinol.

[69] Basnet S, Kozikowski A, Sun H, Troup M, Urrutia LE, Pekmezaris R (2017). Metformin therapy and postoperative atrial fibrillation in diabetic patients after cardiac surgery. J Intensive Care.

[70] Al-Khatib SM, Stevenson WG, Ackerman MJ, Bryant WJ, Callans DJ, Curtis AB, Deal BJ, Dickfeld T, Field ME, Fonarow GC (2018). 2017 AHA/ACC/HRS guideline for management of patients with ventricular arrhythmias and the prevention of sudden cardiac death: executive summary: a Report of the American College of Cardiology/American Heart Association Task Force on Clinical Practice Guidelines and the Heart Rhythm Society. Heart Rhythm.

[71] Prenner SB, Shah SJ, Goldberger JJ, Sauer AJ (2016). Repolarization heterogeneity: beyond the QT interval. J Am Heart Assoc.

[72] Nishiyama A, Ishii DN, Backx PH, Pulford BE, Birks BR, Tamkun MM (2001). Altered K(+) channel gene expression in diabetic rat ventricle: isoform switching between Kv4.2 and Kv1.4. Am J Physiol Heart Circ Physiol.

[73] Shimoni Y, Severson D, Ewart HS (2000). Insulin resistance and the modulation of rat cardiac K(+) currents. Am J Physiol Heart Circ Physiol..

[74] Yada H, Murata M, Shimoda K, Yuasa S, Kawaguchi H, Ieda M, Adachi T, Murata M, Ogawa S, Fukuda K (2007). Dominant negative suppression of Rad leads to QT prolongation and causes ventricular arrhythmias via modulation of L-type Ca2 + channels in the heart. Circ Res.

[75] Brown DW, Giles WH, Greenlund KJ, Valdez R, Croft JB (2001). Impaired fasting glucose, diabetes mellitus, and cardiovascular disease risk factors are associated with prolonged QTc duration. Results from the Third National Health and Nutrition Examination Survey. J Cardiovasc Risk.

[76] Christensen PK, Gall MA, Major-Pedersen A, Sato A, Rossing P, Breum L, Pietersen A, Kastrup J, Parving HH (2000). QTc interval length and QT dispersion as predictors of mortality in patients with non-insulin-dependent diabetes. Scand J Clin Lab Invest.

[77] Sawicki PT, Kiwitt S, Bender R, Berger M (1998). The value of QT interval dispersion for identification of total mortality risk in non-insulin-dependent diabetes mellitus. J Intern Med.

[78] Costa EC, Goncalves AA, Areas MA, Morgabel RG (2008). Effects of metformin on QT and QTc interval dispersion of diabetic rats. Arq Bras Cardiol.

[79] Wang H, Wang C, Lu Y, Yan Y, Leng D, Tian S, Zheng D, Wang Z, Bai Y (2020). Metformin shortens prolonged QT interval in diabetic mice by inhibiting L-type calcium current: a possible therapeutic approach. Front Pharmacol.

[80] Lv L, Zheng N, Zhang L, Li R, Li Y, Yang R, Li C, Fang R, Shabanova A, Li X (2020). Metformin ameliorates cardiac conduction delay by regulating microRNA-1 in mice. Eur J Pharmacol.

[81] Danik SB, Rosner G, Lader J, Gutstein DE, Fishman GI, Morley GE (2008). Electrical remodeling contributes to complex tachyarrhythmias in connexin43-deficient mouse hearts. FASEB J.

[82] Poelzing S, Rosenbaum DS (2004). Altered connexin43 expression produces arrhythmia substrate in heart failure. Am J Physiol Heart Circ Physiol.

[83] Roell W, Lewalter T, Sasse P, Tallini YN, Choi BR, Breitbach M, Doran R, Becher UM, Hwang SM, Bostani T (2007). Engraftment of connexin 43-expressing cells prevents post-infarct arrhythmia. Nature.

[84] Surinkaew S, Kumphune S, Chattipakorn S, Chattipakorn N (2013). Inhibition of p38 MAPK during ischemia, but not reperfusion, effectively attenuates fatal arrhythmia in ischemia/reperfusion heart. J Cardiovasc Pharmacol.

[85] Jackson PE, Feng QP, Jones DL (2008). Nitric oxide depresses connexin 43 after myocardial infarction in mice. Acta Physiol (Oxf).

[86] Yin M, van der Horst IC, van Melle JP, Qian C, van Gilst WH, Sillje HH, de Boer RA (2011). Metformin improves cardiac function in a nondiabetic rat model of post-MI heart failure. Am J Physiol Heart Circ Physiol.

[87] Calvert JW, Gundewar S, Jha S, Greer JJ, Bestermann WH, Tian R, Lefer DJ (2008). Acute metformin therapy confers cardioprotection against myocardial infarction via AMPK-eNOS-mediated signaling. Diabetes.

[88] Lu L, Ye S, Scalzo RL, Reusch JEB, Greyson CR, Schwartz GG (2017). Metformin prevents ischaemic ventricular fibrillation in metabolically normal pigs. Diabetologia.

[89] Weidner K, Behnes M, Schupp T, Rusnak J, Reiser L, Bollow A, Taton G, Reichelt T, Ellguth D, Engelke N (2018). Type 2 diabetes is independently associated with all-cause mortality secondary to ventricular tachyarrhythmias. Cardiovasc Diabetol.

[90] Marfella R, Rossi F, Giugliano D (1999). QTc dispersion, hyperglycemia, and hyperinsulinemia. Circulation.

[91] Tran HV, Gore JM, Darling CE, Ash AS, Kiefe CI, Goldberg RJ (2018). Hyperglycemia and risk of ventricular tachycardia among patients hospitalized with acute myocardial infarction. Cardiovasc Diabetol.

[92] Jardine DL, Charles CJ, Frampton CM, Richards AM (2007). Cardiac sympathetic nerve activity and ventricular fibrillation during acute myocardial infarction in a conscious sheep model. Am J Physiol Heart Circ Physiol.

[93] Najeed SA, Khan IA, Molnar J, Somberg JC (2002). Differential effect of glyburide (glibenclamide) and metformin on QT dispersion: a potential adenosine triphosphate sensitive K + channel effect. Am J Cardiol.

[94] Cacciapuoti F, Spiezia R, Bianchi U, Lama D, D’Avino M, Varricchio M (1991). Effectiveness of glibenclamide on myocardial ischemic ventricular arrhythmias in non-insulin-dependent diabetes mellitus. Am J Cardiol.

[95] Targeting Risk Interventions and Metformin for Atrial Fibrillation (TRIM-AF). https://ClinicalTrials.gov/show/NCT03603912. 10.1007/s10840-024-01955-z PMC11832320 39671157

[96] Metformin as an Upstream Therapy in Atrial Fibrillation. https://ClinicalTrials.gov/show/NCT02931253.

